# Thermoelectric properties of doped graphene nanoribbons: density functional theory calculations and electrical transport

**DOI:** 10.1039/d1ra08303a

**Published:** 2022-02-21

**Authors:** E. Rahmati, A. Bafekry, M. Faraji, D. Gogva, Chuong V. Nguyen, M. Ghergherehchi

**Affiliations:** School of Physics, University of Damghan P.O. Box 36716-41167 Damghan Iran; Department of Physics, University of Guilan Rasht 41335-1914 Iran bafekry@.asad@gmail.com; Micro and Nanotechnology Graduate Program, TOBB University of Economics and Technology Sogutozu Caddesi No. 43 Sogutozu 06560 Ankara Turkey; Department of Physics, Chemistry and Biology, Linkoping University 58183 Linkoping Sweden; Department of Materials Science and Engineering, Le Quy Don Technical University Hanoi Vietnam; Department of Electrical and Computer Engineering, Sungkyunkwan University 16419 Suwon Korea mitragh@skku.edu; Department of Physics, Rasht Branch, Islamic Azad University Rasht Iran

## Abstract

We present a detailed study on band structure-dependent properties such as electrical conductivity, the charge of carriers and Seebeck coefficients of graphene nano-ribbons (GNRs) doped with the magnetic impurities Fe and Co since the spin thermopower could be considerably enhanced by impurities. Thermoelectric properties of two-dimensional systems are currently of great interest due to the possibility of heat to electrical energy conversion at the nanoscale. The thermoelectric properties are investigated using the semi-classical Boltzmann method. The electronic band structure of doped nano-ribbons is evaluated by means of density-functional theory in which the Hubbard interaction is considered. Different types of nano-ribbons (armchair-edge and zigzag-edge) and their thermoelectric features such as conductivity and Seebeck coefficient in the presence and absence of magnetic impurities have been studied.

## Abstract 2

We present a detailed study on band structure-dependent properties such as electrical conductivity, the charge of carriers and Seebeck coefficients of graphene nano-ribbons doped with atoms since the spin thermopower could be enhanced by impurities.

## Graphical abstract

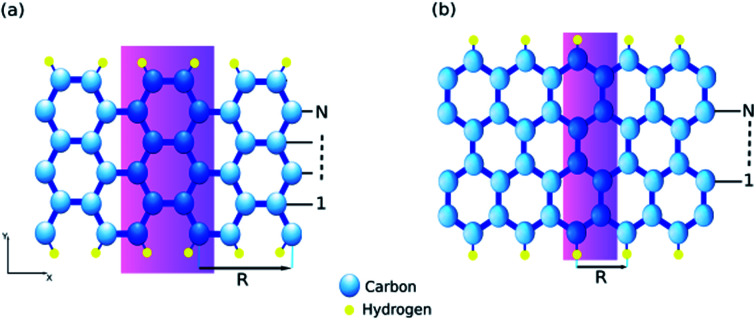

## 1 Introduction

After the discovery of graphene^1^ as the first two-dimensional (2D) material, huge attention has been attracted to the search for other 2D graphene-like materials. The last have demonstrated exceptional physical and chemical properties which can be utilized in next generation electronics and optoelectronics. Recently, due to their unique properties, graphene and other 2D graphene-driven nanosheets have been studied both experimentally and theoretically by means of density functional theory (DFT).^2–9^ The thermoelectric, mechanical, and optical properties of graphene and other graphene-like 2D nanosheets can be promoted by different processes, including doping with other elements as an impurity,^10^ making ribbons^11^ and heterostructures,^12^ creating atomic vacancies,^13^ and so on. One-dimensional structure of graphene namely graphene nanoribbons (GNRs) are thin ribbons of graphene that have brought additional benefits over graphene monolayer.^14^ For instance, GNRs have a higher aspect ratio with respect to graphene, which importantly decreases the percolation threshold in polymer composites and conductive films. Two types of nanoribbons can be derived from the graphene monolayer, namely armchair graphene nanoribbons (AGNRs) and zigzag graphene nanoribbons (ZGNRs). The GNRs are perfect for nano-electronic applications,^15–20^ and numerous researches have been implemented on the electronic properties of GNRs by different methods, including DFT calculations, non-equilibrium Green's function method, mean field theory, and tight-binding calculations.^21^

The thermoelectric properties arise from a thermoelectric material with the ability to generate electricity from temperature gradients. The Peltier's and Seebeck's effects are the most interesting aspect of materials with thermoelectric properties. The efficiency of a thermoelectric material can be expressed by the power factor, which is one of the characteristics of materials to determine the utility of a thermoelectric cooler or generator. To predict a sufficiently high thermoelectric performance, phenomenologically, calculating the band structure-dependent quantities are needed.^22^ However, the thermal properties of GNRs structure have been studied for thermoelectric applications,^23^ some researches have revealed that it is possible to promote thermoelectric properties of GNRs by modifying the atomic configuration of the ribbon's edge.^24,25^ Recently, the GNRs junction structures and their thermoelectric features have been studied.^26^ In another study, the electronic transport characteristics of GNRs have been simulated by means of nearest-neighbor π-orbital tight-binding Hamiltonian, and the thermal transport has been modeled by the forth-nearest-neighbor force constant model (4-NNFC).^27,28^

Recently, a lot of efforts have been carried out to modify the electronic and magnetic properties of graphene to make it useful for realistic applications. The gapless nature of graphene can be changed by adding a small number of heteroatoms^29^ and the characteristics of graphene can be tuned by dopping impurities by substitutional or adsorbing atoms.^30–33^

The effect of impurities on graphene have been studied theoretically using different methods such as tight binding and Green's function methods.^34^ Mean-field approximation has been used to model the magnetic impurities through Hubbard term.^35,36^ It has been demonstrated that cobalt dopping can be used in band gap controlling in graphene.^37^

In this paper, the thermoelectric properties of doped GNRs have been studied in detail. For example, in ZGNRs as a host, the configuration of the edges play a pivotal role in conductivity, and the magnetic impurities will change the energy bands of the host dramatically due to the Hubbard effect.^38^ Understanding the physics of the localized spins on a metal host is significant for the engineering of doped thermoelectric nanoscale devices. Here, the behavior of thermoelectric features around the Fermi level has been investigated using Boltzmann's method.

## 2 Methodology

**Fig. 1:**
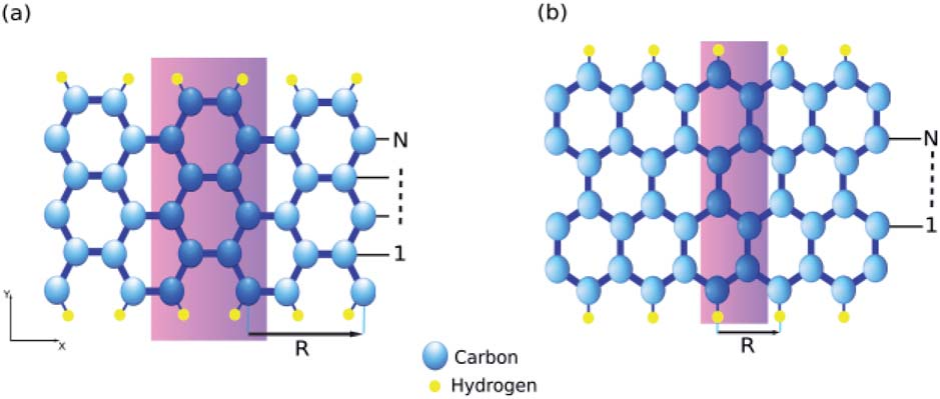
Structure of graphene nano-ribbon with (a) armchair edges (armchair-edge graphene nano-ribbon) and (b) zigzag edges (zigzag graphene nano-ribbon). The lattice constant is **R** and **N** defines the nano-ribbon width. The yellow circles indicate the hydrogen atoms for the edge boundary condition of mass-less Dirac equation. Note, the passivated carbons are excluded from the labels since they do not contribute in the conduction.

There are two typical kinds of edges in graphene nanoribbons called armchair-edge and zigzag-edge. The two edges have 30° difference in their cutting direction. Here we briefly discuss the structure and the procedure of calculating band-structure dependent properties. We have considered our system as the one illustrated in Fig. 1. Following a common agreement, we demonstrate both ZGNRs and AGNRs by the number of dimers (two carbon sites) *N* in the unit cell which is shown as ZGNR(*N*) and AGNR(*N*). Atomic structure relaxations were performed using the linear combination of pseudo-atomic orbitals (LCPAO) method within the quasi-Newton scheme till the forces on the atoms become less than 10^−5^ eV Å^−1^. The optimized lattice constant and atomic positions for both AGNR (*a* = 4.28 Å) and ZGNR (*a* = 2.47 Å) are in agreement with some previous works.^39,40^ The exchange-correlation energy was taken into account by using the local density approximations (LDA) and a kinetic cutoff energy of 400 eV for the plane-wave basis was adopted. A Monkhorst–Pack mesh (12 × 1 × 1) was set for the Brillouin zone.

**Fig. 2:**
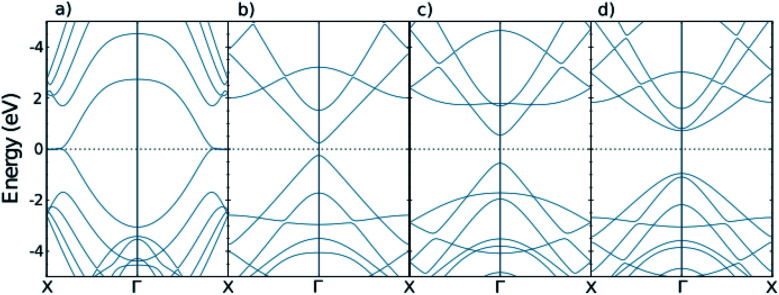
The band structures of pure nano-ribbons. Respectively, (a)–(d) are related to ZGNR(3), AGNR(3), AGNR(4) and AGNR(5).

It has been taken into account that all suspended bonds at graphene edges are terminated by hydrogen atoms, and thus give no contribution to the electronic states near the Fermi level. The band-structures of both systems are displayed in Fig. 2. Our calculations are in agreement with previous works.^41^ Based on the ref. 41 AGNRs can be classified into three groups as AGNR with *N* = 3*n*, *N* = 3*n* + 1, and *N* = 3*n* + 2 dimer lines. It is essential to study three groups since AGNRs show a periodic behavior in band gap and other AGNRs with higher width show a similar characteristics.

In this section, we are inspired by the semi-classical Boltzmann approximation used in quasi-one-dimensional GNRs. We calculate the conductivity employing the semi-classical motion of the electron in the presence of gradient and disturbing fields. To describe the conduction, we use the equilibrium Dirac distribution function *f*(*ε*). Based on the Boltzmann theory the conductivity is obtained as follows^42^1
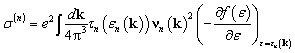
where *τ*_*n*_(*ε*_*n*_(**k**)) is the relaxation time which is energy dependent and can be evaluated from the following equation^42^2
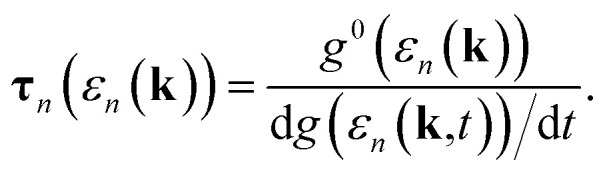
and *ν*_*n*_(**k**) is the semi-classical velocity of electron defined as a function of band energies as follows3
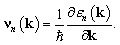


Note that we need the total conductivity *σ*, which is a sum of contributions from each band4
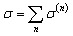


Using the definition of the conversion of heat directly into electricity or *vice versa* the Seebeck coefficient can be written as follows:

5 *S* = *σ*^−1^**ν**_*n*_(**k**).

In the Boltzmann theory, the average free time of flight of a charge carrier is defined as relaxation time *τ* which is inversely proportional to the scattering probability of the charge carrier from atoms. *τ* is closely related to the electrical conductivity and other thermoelectric properties of the system. Furthermore, the charge of carriers can be evaluated from the density of states (DOS) *g*(*ε*) calculated by DFT. One can calculate the charge of carriers as follows:

6 *n* = *e*∫*g*(*ε*)*f*(*ε*)d*ε*.

Thermoelectric properties of the system have been calculated using the BoltzTraP.^43^ Calculating the Boltzmann conductivity needs to evaluate band-structures and energies in which we will utilize the OpenMX software package based on DFT. Atomic structure relaxations and electronic properties calculations were performed employing the OpenMX package^44^ within the linear combination of pseudo-atomic orbitals (LCPAO) method.^45^

## 3 Electronic properties of doped GNRs

**Fig. 3:**
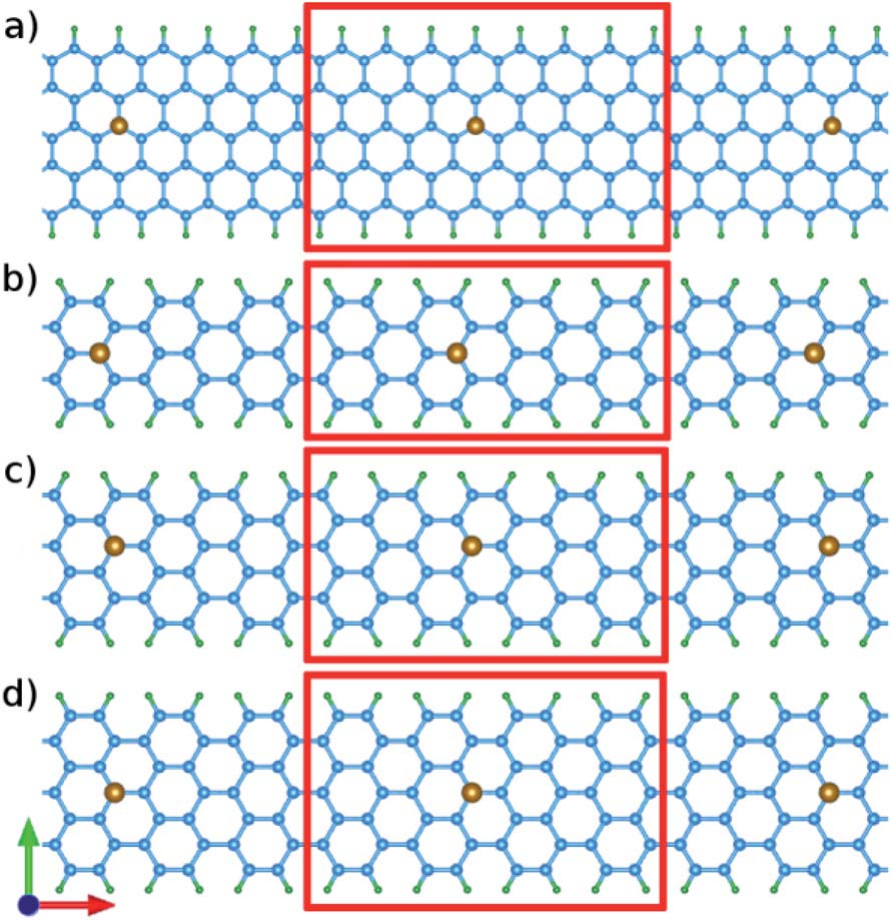
Structure of doped ZGNR(3) (a), AGNR(3) (b), AGNR(4) (c) and AGNR(5) (d) with magnetic impurities. The blue and green atoms are carbon and hydrogen atoms respectively. The brown atoms stand for Fe or Co atoms as magnetic impurities. The red box indicates the unit-cell considered in DFT calculations. The red, green and blue arrows are **a**, **b** and **c** lattice vectors, respectively.

We studied iron (Fe) and cobalt (Co) as magnetic impurities in which the Hubbard interaction plays an important role in the electronic and spintronic properties of the system. The structure used in DFT calculations is presented in Fig. 3. Respectively, Fig. 3(a)–(d) show the doped ZGNR(3), AGNR(3), AGNR(4) and AGNR(5). After the structure optimization, the lattice constant of the ZGNR and AGNR was determined as: 19.80 Å and 17.12 Å. These lattice vectors guaranty iron and cobalt atoms to be far from each other and to act as impurities. As shown in Fig. 3, it was assumed that the impurities are located in the centre of the ribbon approximately.

As mentioned before, the Hubbard interaction plays an important role in magnetic systems. So, we should find the Hubbard correction (*U*_eff_) for doped GNRs. To achieve this goal we should consider DFT + U corrections in the first-principles calculations^46,47^ because the on-site Coulomb interactions are not correctly described by the LDA or GGA when we have magnetic impurities. The idea behind the DFT + U is to correct the strong on-site Coulomb interaction of the electrons which are localized with an additional Hubbard term. To increase the accuracy of the Brillouin zone integrations the Monkhorst–Pack mesh is changed to (15 × 1 × 1).

**Fig. 4:**
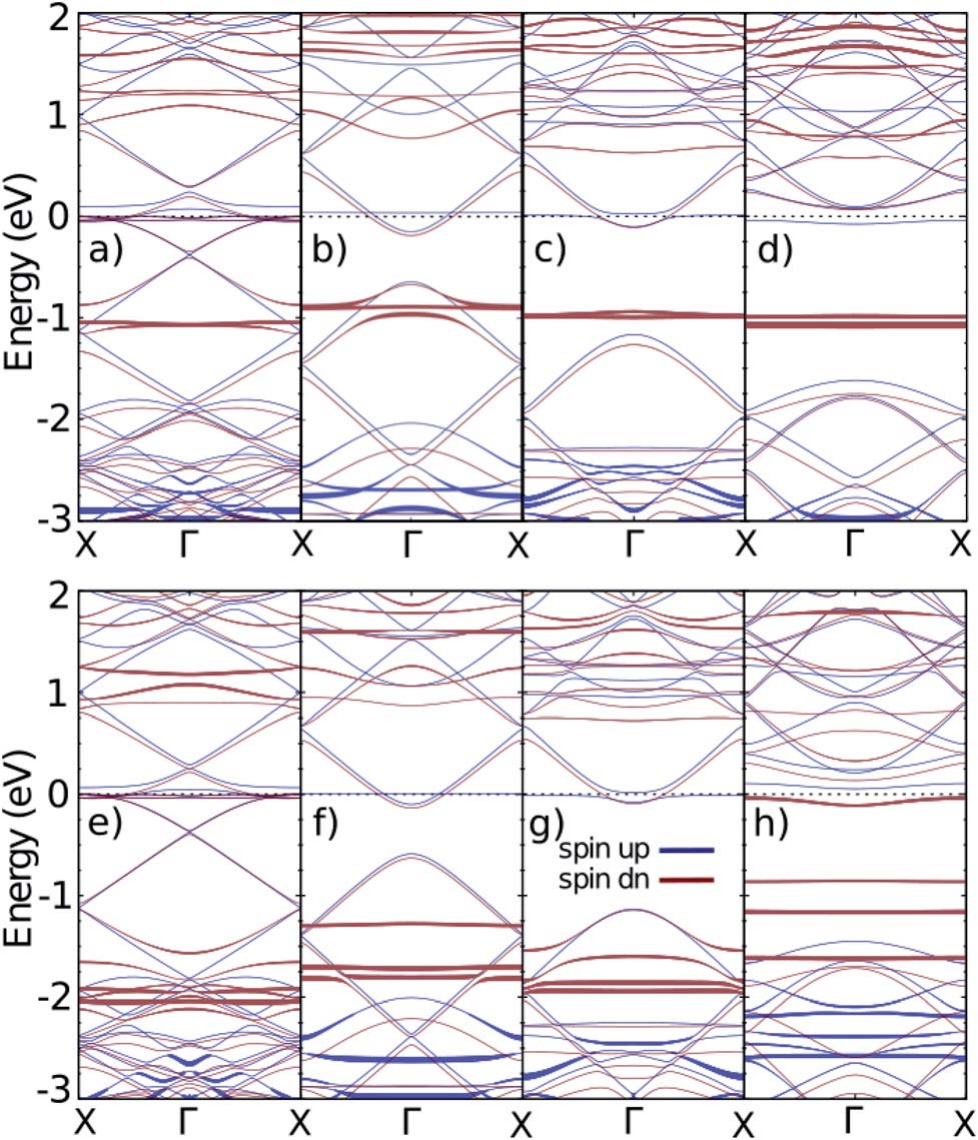
The band structures of nano-ribbons with impurities. (a)–(d) are related to iron doped ZGNR(3), AGNR(3), AGNR(4) and AGNR(5) and (e)–(h) are related to cobalt doped ones. The thickness of the bands shows the contribution of d-orbitals of the impurities.

Doping with magnetic impurities changes the band structures of ZGNRs and AGNRs. As revealed in Fig. 4, doping with iron and cobalt have different influence on the band structures of GNRs. The flat bands in the top and bottom figures are related to the energy levels of iron and cobalt, respectively. The bandwidth indicates the contribution of spin up and down of different orbitals in different band numbers and wave vectors.

Both iron and cobalt have an energy level near the Fermi level. The separation of the spin up and down in AGNR(3*n*) and AGNR(3*n* + 1) are considerable because only the spin-down band remains near the Fermi level. Although the flat bands do not contribute to the conductivity because of localized electrons around the impurities, they affect the magnetic features of the system. The electrons with zero velocity localized in impurities' orbitals can feed the non-flat states of carbon atoms in the GNRs. Because the electrons in the edge sites contribute to conductivity, the distance of the impurities from the edge of the ribbon plays an essential role in its electronic behavior. The carbon bands near the Fermi level are related to the edge state and so the width of ribbons will change the results of separation of spin up and down. That is why the separation between spin up and down of the flat bands in AGNR(3*n* + 2) is less than the others.

Doping magnetic impurities on the host semiconductors induces spin polarization in the band gap of the system. The d-orbital electrons play an important role in magnetic properties of the doped sample.^48^ Since the source of magnetic properties of Fe and Co is their d-orbitals, we show the contribution of d-orbitals of the impurities are illustrated in Fig. 4. The band gaps for pure and doped AGNRs are compared in Table 1. Note that the energy states introduced in the band gap which are sourced from the impurities are dopant states and these states should not be considered as conduction or valence band. The band gaps in Fe-doped AGNRs are less than pure ones, while band gaps of Co-doped AGNRs are larger than pure samples.

**Table 1:** The band gaps of the pure and doped AGNRs in electron-volt

Width sample	Pure	Fe-doped	Co-doped
AGNR(3)	0.53	0.49	1.05
AGNR(4)	1.12	0.49	1.82
AGNR(5)	1.94	0.83	1.66

From the band structures we can categorize the systems into three groups. ZGNRs are Dirac semiconductors, Fig. 4(f) and (g) are normal semiconductors and n-type or p-type semiconductors. (c), (d), and (h) are p-type semi conductor because the flat band is near to valence band of AGNRs. For example, in AGNR(3*n* + 1) and AGNR(3*n* + 2) Fe atoms play as electron donor dopant which can releases a mobile conduction electron into the crystal lattice if the applied voltage is higher than cut voltage. It should be noted that the donor levels are spin-down electrons. However, it is possible to control the properties of all these categories using an external magnetic field.^49^

It has been also reported that iron impurity acts as a donor in Bi_2_Te_3_, Sb_2_Te_3_ and Bi_2_Se_3_ single crystals.^50^ Furthermore, in the case of AGNR(3*n* + 2) one can find out that the spin-up and spin-down electrons show different behaviours in flat bands. The dopant states are made of spin-down electron and the spin-up band-structure is a normal semiconductor without any dopant states.

## 4 Thermoelectric properties of pure and doped GNRs

In this section, we present the thermoelectric properties of graphene nanoribbons using first-principle calculations. Using BoltzTraP code *via* an interface to OpenMX,^51^ one can calculate the thermoelectric properties of such a system. Semi-classical transport coefficients such as Seebeck coefficient and electrical conductivity were calculated under the constant relaxation time at temperature 300 K. Semi-classical transport features are presented as a function of chemical potential (*μ*) which is an independent variable. Substitution and doping can be used to manipulate *μ* which plays an important role in the thermoelectric transport properties.^52^

**Fig. 5:**
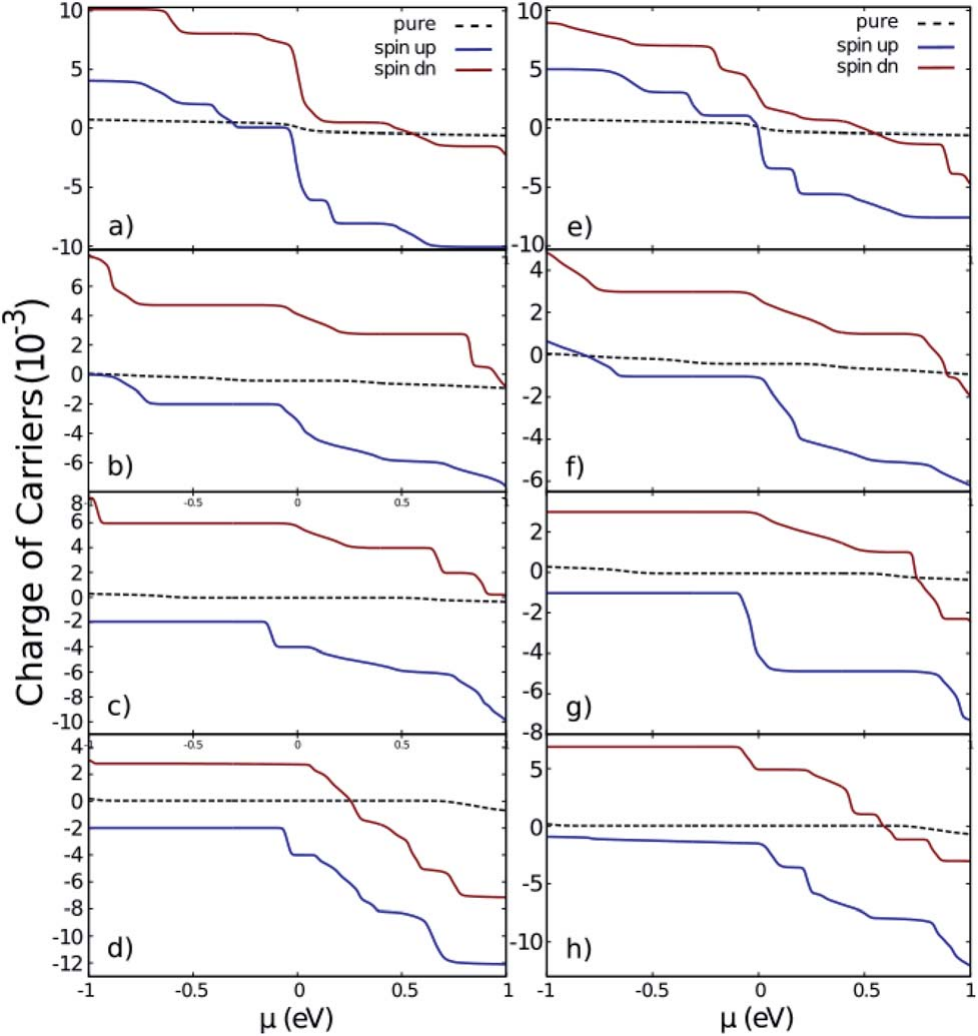
The charge of carriers of nano-ribbons with impurities. (a)–(d) are related to iron doped ZGNR(3), AGNR(3), AGNR(4) and AGNR(5) and (e)–(h) are related to cobalt doped ones. Black dashed lines correspond to the pure nano-ribbons calculated from the band-structures depicted in Fig. 2.

Fig. 5 displays the charge of carriers (*n*) in terms of chemical potential in different cases. In this figure, we compare the doped GNRs (coloured solid lines) with pure GNRs (dashed black lined). The charge of carriers in the case of doped GNRs increased dramatically in comparison with the pure GNRs. This is completely the correct behaviour of a doped crystal. The current of the GNRs can be evaluated as the product of the average velocity calculated from band structure times the corresponding charge of carriers. Fig. 5 indicates that in the iron and cobalt doped AGNRs the majority carriers near on the top of the Fermi level for the spin up and down are electrons holes, respectively.

**Fig. 6:**
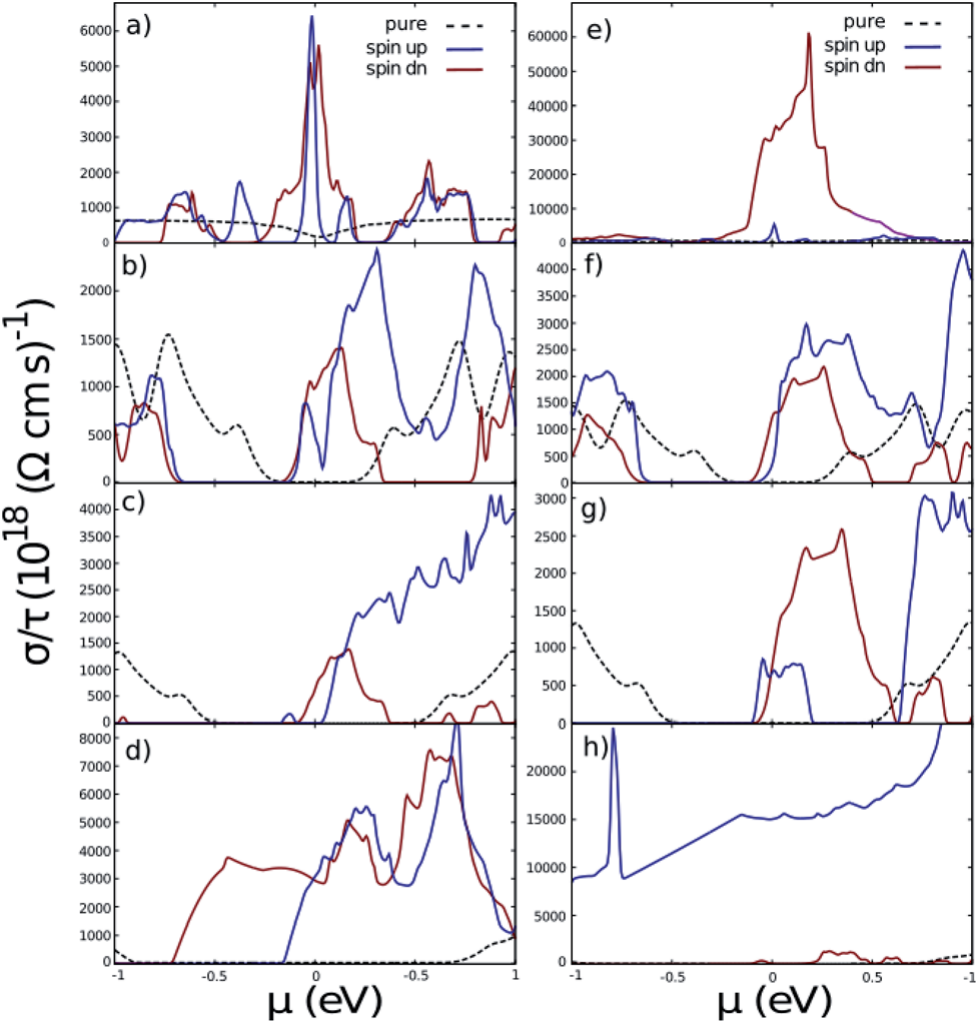
The conductivity per relaxation time of nano ribbons with impurities. (a)–(d) are related to iron doped ZGNR(3), AGNR(3), AGNR(4) and AGNR(5) and (e)–(h) are related to cobalt doped ones. Black dashed lines are related to the pure nano-ribbons calculated from the band-structures exhibited in Fig. 2.

Also, conductivity *σ*/*τ*, is illustrated in Fig. 6, which shows how the symmetry between negative and positive energies is broken after doping because of the Hubbard interaction. There is a dramatical difference between spin up and down in conductivity in the case of cobalt doped GNRs. Fig. 6(b) and (f) represent similar behavior in conductivity for both spin up and down, but the entire conductivity of cobalt doped AGNR is approximately 2 times of the iron doped one.

**Fig. 7:**
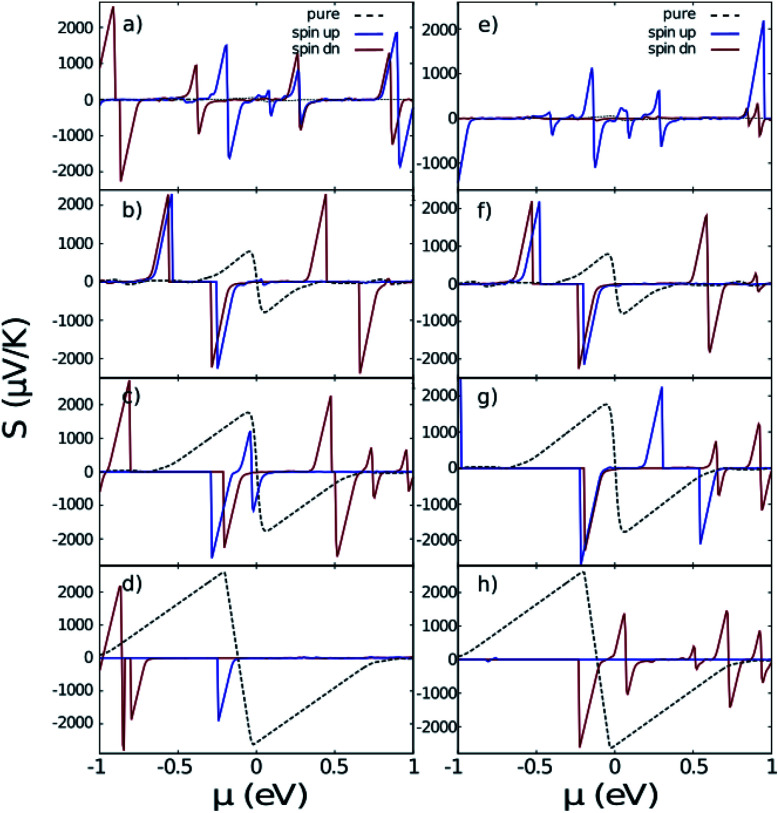
The Seebeck coefficient of nano-ribbons with impurities. (a)–(d) are related to iron doped ZGNR(3), AGNR(3), AGNR(4) and AGNR(5) and (e)–(h) are related to cobalt doped ones. Black dashed lines correspond to the pure nano-ribbons calculated from the band-structures shown in Fig. 2.

Fig. 7 indicates the Seebeck coefficient *S* in terms of chemical potential. It is noticeable that like conductivity, the symmetry between negative and positive energies is broken after taking the Hubbard interaction into account. The most important fact evidenced from Fig. 7 is that the Seebeck coefficient of the doped systems is larger than that of the pure ones in the case of ZGNR and AGNR with *N* = 3*n*. Meanwhile, the Seebeck coefficient of AGNRs with *N* = 3*n* + 1 and *N* = 3*n* + 2 decreases with doping.

Concerning cobalt doped GNRs, the Seebeck coefficient of spin-up ZGNR is dominant in the whole range of chemical potential, but in the case of AGNR *N* = 3*n* + 2 the Seebeck coefficient of spin-up is negligible. In other words, Co-doped ZGNR and AGNR(5) are good candidates for filtering spin-down and spin-up electrons respectively.

## 5 Conclusion

Band structure-dependent properties of graphene nanoribbons such as the charge of carrier, conductivity and Seebeck coefficients have been investigated by a powerful combination of the semi-classical Boltzmann theory and DFT. The properties of GNRs doped with application-relevant magnetic impurities are studied in detail and compared with those of pure ones. We studied the effect of magnetic impurities *i.e.* Fe and Co on the electronic structure of GNRs with different widths. The energy states of the impurities can act as dopant states in AGNRs. Besides, the Seebeck coefficient of the doped nano-ribbons shows that the Co-doped ZGNR and AGNR (3*n* + 2) are two candidates for spin filter heat to electricity conversion.

## Data availability

The data that support the findings of this study are available from the corresponding author upon reasonable request.

## Conflicts of interest

The authors declare that there are no conflicts of interest regarding the publication of this paper.

## Supplementary Material
